# Cost-effectiveness of alternative strategies for vaccination of adolescents against serogroup B IMD with the MenB-FHbp vaccine in Canada

**DOI:** 10.17269/s41997-019-00275-4

**Published:** 2020-01-06

**Authors:** Marie-Claude Breton, Liping Huang, Sonya J. Snedecor, Noelle Cornelio, Fiorella Fanton-Aita

**Affiliations:** 1 Pfizer Canada ULC, 17300 Trans-Canada Highway, Kirkland, Montréal, QC H9J 2M5 Canada; 2 Pfizer Inc., Collegeville, PA USA; 3 Pharmerit International, Bethesda, MD USA

**Keywords:** Cost-effectiveness analysis, Meningococcal vaccine, Meningococcal disease, Canada, Adolescents, Transmission dynamic model, Economic model, Analyse coût-efficacité, Vaccin méningococcique, Méningococcie, Canada, Adolescents, Modèle dynamique de transmission, Modèle économique

## Abstract

**Objective:**

Serogroup B meningococci (MnB) are now the largest cause of invasive meningococcal disease (IMD) in Canada. We assessed the clinical and economic impact of 3 adolescent MenB-FHbp immunization strategies.

**Methods:**

A population-based dynamic transmission model was developed to simulate the transmission of MnB among the entire Canadian population over a 30-year time horizon. Age group-based IMD incidence, bacterial carriage and transmission, disease outcomes, costs, and impact on quality of life were obtained from Canadian surveillance data and published literature. The vaccine was assumed to provide 85% protection against IMD and 26.6% against carriage acquisition. The model estimated the impact of routine vaccination with MenB-FHbp in 3 strategies: (1) age 14, along with existing school-based programs, with 75% uptake; (2) age 17 with 75% uptake, assuming school vaccination; and (3) age 17 with 30% uptake, assuming vaccination outside of school. Costs were calculated from the Canadian societal perspective.

**Results:**

With no vaccination, an estimated 3974 MnB cases would be expected over 30 years. Vaccination with strategies 1–3 were estimated to avert 688, 1033, and 575 cases, respectively. These outcomes were associated with incremental costs per quality-adjusted life-year of $976,000, $685,000, and $490,000.

**Conclusions:**

Our model indicated that if the vaccine reduces risk of carriage acquisition, vaccination of older adolescents, even at lower uptake, could have a significant public health impact. Due to low disease incidence, MnB vaccination is unlikely to meet widely accepted cost-effectiveness thresholds, but evaluations of new programs should consider the overall benefits of the vaccination.

## Résumé

**Objectif:**

Le méningocoque du sérogroupe B (MnB) représente aujourd’hui la cause la plus importante de méningococcie invasive au Canada. Cette analyse a évalué les répercussions cliniques et économiques de 3 stratégies d’immunisation à l’aide du vaccin contre le MenB-fHBP chez des adolescents.

**Méthodes:**

Un modèle dynamique de transmission dans la population a été créé afin de simuler la propagation du MnB dans l’ensemble de la population canadienne pendant une période de 30 ans. L’incidence de la méningococcie invasive par groupe d’âge, les taux de portage et de transmission bactérienne, l’évolution de la maladie, les coûts qui y sont associés et les répercussions sur la qualité de vie ont été tirés de rapports de surveillance canadiens et de la littérature. Il a été supposé que le vaccin offrait une protection contre la méningococcie invasive et contre l’acquisition du portage dans 85 % et 26,6 % des cas, respectivement. Le modèle étudié a évalué les répercussions associées à l’inoculation systématique par le vaccin contre le MenB-fHBP selon les 3 stratégies suivantes : (1) immunisation à l’âge de 14 ans, dans le cadre des programmes existants mis en œuvre en milieu scolaire, à un taux de vaccination de 75 %; (2) immunisation à l’âge de 17 ans, en milieu scolaire, à un taux de vaccination de 75 %; et (3) immunisation à l’âge de 17 ans, à un taux de vaccination de 30 %, en contexte extrascolaire. Les coûts ont été calculés en fonction de la perspective sociétale canadienne.

**Résultats:**

Sans immunisation, le nombre estimé de cas de méningococcie du sérogroupe B serait de 3974 sur une période de 30 ans. Selon le modèle, les stratégies de vaccination 1 à 3 préviendraient respectivement 688, 1033 et 575 cas. Ces résultats ont été associés à des coûts différentiels par année de vie gagnée ajustée par la qualité (QALY) de 976 000 $, 685 000 $ et 490 000 $.

**Conclusions:**

Le modèle utilisé dans le cadre de l’analyse a permis d’indiquer que si le vaccin réduisait le risque d’acquisition du portage, la vaccination des adolescents plus vieux, même en présence de taux de vaccination plus faibles, pouvait avoir des répercussions significatives sur la santé publique. En raison de la faible incidence de la maladie, il est peu probable que la vaccination contre le MnB respecte les seuils généralement acceptés en matière de coût-efficacité, mais l’évaluation de nouveaux programmes devrait tenir compte de l’ensemble des bienfaits associés à la vaccination.

## Introduction

Invasive meningococcal disease (IMD) is a rare but serious condition caused by *Neisseria meningitidis*, with potentially devastating consequences. The bacteria can be present on the nasopharyngeal mucosa without causing the disease but, in a small proportion of carriers, will invade the meninges or blood, leading to meningitis and/or septicemia (Crum-Cianflone and Sullivan 2016). The onset of IMD can be insidious and the early manifestations are often indistinguishable from a number of other mostly benign infections. IMD can be fatal within 24 h of onset (Public Health Agency of Canada 2015; World Health Organization 2018). Up to one third of survivors will have permanent severe sequelae such as hearing loss, neurological disabilities, or limb loss (Bettinger et al. 2013; Sadarangani et al. 2015; Public Health Agency of Canada 2015; Ontario Ministry of Health and Long-Term Care 2015). While disease incidence is highest in infants and children under 5 years of age, a significant number of cases occur in adolescents and young adults (National Advisory Committee on Immunization 2014)—the population segment in whom meningococcal carriage prevalence is highest (Christensen et al. 2010).

IMD is endemic in Canada and the majority of cases are caused by serogroup B (MnB) (National Advisory Committee on Immunization 2014; Li et al. 2014), including the recent outbreak in Nova Scotia, as well as the prolonged increased incidence in the province of Quebec (De Wals et al. 2017; Langley et al. 2016; Nova Scotia Department of Health and Wellness 2015). Serogroup B meningococcus was responsible for 63% of all IMD cases in Canada between 2011 and 2015 (Public Health Agency of Canada 2017). In October 2017, Health Canada approved the MenB-FHbp vaccine (Trumenba®, Pfizer) for use in individuals aged 10 through 25 years (Pfizer Canada Inc. 2018). Before then, the multicomponent MnB (4CMenB) vaccine (Bexsero®, Novartis), approved in December 2013, was the only MnB vaccine available in Canada (GlaxoSmithKline Inc. 2018).

To prevent IMD caused by serogroups A, C, Y, and W-135, infant and adolescent routine immunization programs are in place across Canada (Public Health Agency of Canada 2018). For protection against IMD caused by serogroup B, the National Advisory Committee on Immunization (NACI) recommends^1^ 4CMenB vaccination for individuals at high risk of meningococcal disease and those who have had a close contact with a case, as well as to control outbreaks (National Advisory Committee on Immunization 2014). MnB vaccination is not currently included in Canada’s routine vaccination schedule but several provinces publicly fund the vaccine for recommended individuals at high risk of meningococcal disease. Adolescents and young adults are at highest risk of *N. meningitidis* carriage and transmission (Kaaijk et al. 2014), so routine MnB vaccination in this population could help reduce the burden of IMD in Canada.

The current model was developed to assess the cost-effectiveness of different strategies for adolescent MenB-FHbp vaccination in Canada.

## Methods

### Model description

**Fig. 1:**
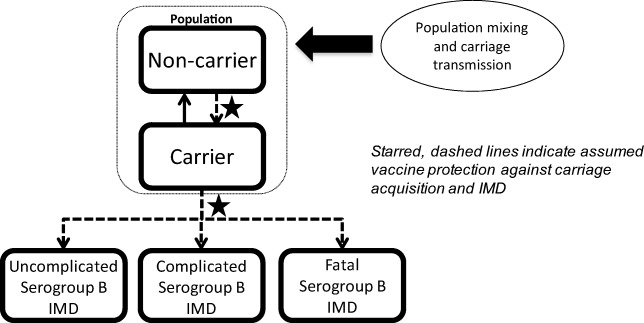
Annual meningococcal carriage and disease model. *IMD* invasive meningococcal disease

A population-based dynamic transmission model was developed to estimate the expected reduction of MnB IMD cases in the 30 years following introduction of routine age-targeted vaccination in the Canadian population. The model structure (Fig. 1) is similar to that published by Ortega-Sanchez et al. (2008). Meningococcal bacteria carriage is the source of infectious transmission and was the primary consideration in the model calculation. The population was stratified into 101 single-year age bands and individuals in each age band transitioned to the next age band in the following year. Each year, the model assumed that a proportion of individuals in each age group were serogroup B *N. meningitidis* carriers who had age-specific probabilities of developing IMD and transmitting the bacteria within their age group or across other age groups (Trotter et al. 2006; Trotter et al. 2002). To calculate meningococcal transmission, the population was stratified into 10 mutually exclusive age groups: 0 to 5 months, 6 to 12 months, 1 year, 2 to 4 years, 5 to 9 years, 10 to 14 years, 15 to 19 years, 20 to 24 years, 25 to 59 years, and ≥ 60 years. During each year in the model’s 30-year time horizon, a proportion of individuals in a targeted age group were vaccinated with MenB-FHbp. The vaccine was assumed to provide direct protection for vaccinated non-carriers against acquiring MnB or for existing carriers against developing IMD. Indirect protection of non-vaccinated individuals due to reduction of carriage prevalence and transmission was also assumed (Marshall et al. 2013; Read et al. 2014). The vaccine’s direct and indirect protection waned as the population aged. Individuals who developed IMD either recovered, with or without complications, or died.

### Model disease inputs

**Fig. 2:**
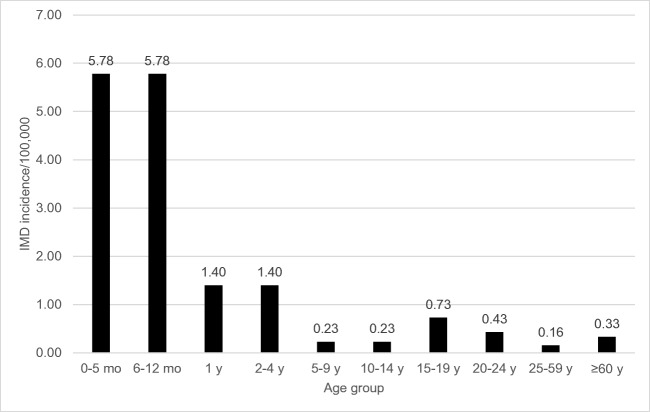
Canadian age-specific incidence for serogroup B IMD (2007–2011 average). *IMD* invasive meningococcal disease, *mo* month, *y* year

MnB incidence rates were derived from 2007 to 2011 Canadian national surveillance—the most recent data available at the time of model development (Fig. 2) (National Advisory Committee on Immunization 2014; National Advisory Committee on Immunization 2013). One scenario analysis assumed 20% higher incidence to address potential underestimation of reported IMD cases, and another used age-specific incidence rates in 2011 through 2016 surveillance data provided by the Public Health Agency of Canada reflecting reduced incidence of IMD in Canada (Public Health Agency of Canada n.d.). IMD-related short- or long-term complications included in the model were skin scarring, amputation, paralysis, seizures, hearing loss, neurologic sequelae, or renal failure. The probabilities of these complications were derived from a cohort study of the outcomes of IMD in adults and children in Canada between 2002 and 2014 (Table 1) (Sadarangani et al. 2015).

**Table 1:** IMD complication probabilities (%) by age group (y)

Complication	< 1	1–4^a^	5–14^b^	15–19	20–24	25–59^c^	≥ 60
Scarring	1.96	7.41	7.19	1.53	1.75	2.04	1.61
Amputation	1.96	3.49	4.14	1.53	2.48	1.46	0.00
Paralysis	1.53	0.65	1.09	3.92	1.75	1.83	2.34
Seizure/epilepsy	6.10	1.96	0.00	0.00	0.00	0.58	0.73
Hearing loss	6.10	4.58	4.14	3.27	3.36	1.39	2.34
Neurologic sequelae	1.53	0.00	0.00	4.14	0.88	1.97	1.46
Renal failure	1.09	0.87	1.09	0.87	1.75	2.26	2.34
Death	4.4	4.4	4.4	4.4	8.6	8.6	8.6

*IMD* invasive meningococcal disease, *y* year

^a^Probabilities for age groups 1 year and 2–4 years were the same

^b^Probabilities for age groups 5–9 years and 10–14 years were the same

^c^Probabilities for age groups 25–44 years and 45–59 years were reported. A weighted average was calculated for the 25–59 age group

As there were no published age-specific MnB carriage data for Canada at the time of analysis, age- and serogroup-specific case-to-carrier ratios for a United Kingdom population (Trotter et al. 2006) were adapted and multiplied by Canadian MnB IMD incidence to derive an estimated baseline prevalence of pre-vaccine MnB carriage in Canada. These ratios represent the probability of disease given carriage and were also used to calculate the number of IMD cases per year and per age group based on the number of unprotected individuals and the estimated carriage prevalence after vaccine introduction.

The model population size was based on the annual Canadian population estimates tables for the year 2016 (Statistics Canada 2016b). The life expectancy at each age was obtained from the Canadian life expectancy table, which captures non-IMD deaths (Statistics Canada 2016a).

### Meningococcal carriage transmission

The model’s dynamic population mixing matrix was based on a published MnC model where 98% of meningococcal transmission was contained within a 3-year age band of individuals 1 year older and younger than the affected carrier and the remaining 2% of the transmission was assumed to come from all other ages equally (Trotter et al. 2005).

In each model year, the number of nasopharyngeal meningococcal carriers in each age group was determined by 4 factors: (1) age-specific carriage prevalence in the prior year; (2) transmission of bacteria within and among age groups; (3) proportion of people vaccinated in each age group; and (4) vaccine efficacy against carriage acquisition. For example, in the 2 to 4 years age group, prevalence was calculated as follows: $\mathrm{prevalence}\ {\left(\mathrm{year}\ n\right)}^{2-4\ \mathrm{years}}\sim {\upbeta}_1^{2-4}\times \mathrm{prevalence}{\left(\mathrm{year}\ n-1\right)}^{0-5\ \mathrm{months}}+{\upbeta}_2^{2-4}\times \mathrm{prevalence}{\left(\mathrm{year}\ n-1\right)}^{6-12\ \mathrm{months}}+\cdots +{\upbeta}_{10}^{2-4}\times \mathrm{prevalence}{\left(\mathrm{year}\ n-1\right)}^{\ge 60\ \mathrm{years}}$, where ${\beta}_i^{2-4}$ represents the proportion of carriage in the 2 to 4 years age group originating from age group *i* and $\sum {\beta}_i^{2-4}=1$. Given the model’s assumption of protection against carriage acquisition, age-specific carriage prevalence decreases over time as a function of vaccine uptake, efficacy, and waning.

### Vaccination scenarios

The model examined 3 immunization strategies using MenB-FHbp vaccine: (1) vaccination at age 14 years with 75% vaccine uptake; (2) vaccination at age 17 with 75% uptake; and (3) vaccination at age 17 with 30% uptake. The 75% uptake rate was estimated from immunization rates reported for school vaccination programs in Ontario for the 2012 to 2013 school year (Ontario Agency for Health Protection and Promotion (Public Health Ontario) 2014). The 30% uptake rate for 17-year-olds was an assumption for vaccination outside of a school setting for older adolescents and is consistent with the 34% uptake rate observed among 17- to 20-year-olds during the 2014 Saguenay Lac St-Jean immunization campaign (De Wals et al. 2017). All adolescents in the target groups were assumed to receive 2 doses of the MenB-FHbp vaccine. The possibility of partial vaccination (i.e., missed doses) was not considered in our model. For all scenarios, uptake was considered immediate upon vaccine introduction and constant over the 30-year time horizon.

### Vaccine efficacy

A population-based study of infants in the UK estimated a 2-dose MnB vaccine effectiveness of 82.9% against MnB cases (Parikh et al. 2016), while a UK model estimated 95% MnB vaccine efficacy to prevent IMD among carriers (Christensen et al. 2016). A 75% to 100% seroresponse rate in adolescents was reported in clinical trials of a 2-dose regimen of the MenB-FHbp vaccine (Vesikari et al. 2016). Thus, we assumed a conservative estimate of 85% vaccine efficacy for adolescents (Table 2). We also assumed 26.6% efficacy against carriage acquisition based on published literature (Read et al. 2014). Although some studies have assumed a 10-year duration of efficacy for adolescent meningococcal vaccine protection (Christensen et al. 2010), we conservatively assumed a 5-year duration of vaccine efficacy against IMD, equal to what was observed with one of the conjugate MnACYW-135 vaccines and consistent with MenB-FHbp immunogenicity studies at 4-year follow-up (Cohn et al. 2017; Patton et al. 2017; Vesikari et al. 2017). Vaccine efficacy against IMD was assumed to decrease by 10% per year over the 5-year duration of protection. Vaccine efficacy against carriage acquisition was assumed to wane faster (20% per year) because higher antibody titers are likely necessary to protect against carriage acquisition. After the assumed duration of protection, both disease and carriage protection efficacy became 0% for the remainder of the 30-year horizon.

**Table 2:** Vaccine efficacy parameters

Parameter	Value
Protection against invasive disease	85% (Vesikari et al. 2016)
Protection against carriage	26.6% (Read et al. 2014)
Duration of protection against disease	5 years (Vesikari et al. 2017)
Duration of protection against carriage	5 years (Vesikari et al. 2017)
Annual decrease in protection against disease	10% (assumption)
Annual decrease in protection against carriage	20% (assumption)

### Costs and disutilities

Costs of model parameters were considered from the societal perspective and included medical costs associated with complicated or uncomplicated IMD treatment or death (e.g., hospital costs to treat IMD, costs of prosthesis after amputation) and costs of caregiver time or lost work (e.g., caregiver loss of work, lost future productivity due to death or neurologic sequelae). All costs were inflated to 2015 Canadian dollars and costs and utilities were discounted at 3% annually (Table 3).

**Table 3:** IMD costs and disutilities

Complication	Direct medical costs^a^	Sources	Productivity costs^a^	Sources	Disutility	Sources
Uncomplicated disease	$14,918		$2988	De Wals et al. (2007)	− 0.0317^b^	Ginsberg et al. (2016)
Scarring	$9347	OCCI (2016)	$0	Assumed	− 0.08	Bijlard et al. (2017)
Amputation	$22,070	OCCI (2016)	≤ 24 years: $244,136^c^ 25–59 years: $136,124 ≥ 60 years: $14,493	Rancourt et al. (2003)	− 0.32	Shepard et al. (2005)
Paralysis	$6559	OCCI (2016)	$0	No data available	− 0.32	Assumed equal to amputation
Seizure/epilepsy	$11,028	OCCI (2016)	$0	No data available	− 0.053	Ginsberg et al. (2016)
Hearing loss	$0	No data available	$64,758^d^	Chen et al. (2014)	− 0.033	Ginsberg et al. (2016)
Neurologic sequelae	$8743	OCCI (2016)	≤ 24: $1,220,682^e^ 25–59: $680,621 ≥ 60: $72,463	Shepard et al. (2005)	− 0.56	Oostenbrink et al. (2002)
Renal failure	$12,047	OCCI (2016)	$0	No data available	− 0.107	Ginsberg et al. (2016); Murray and Lopez (1996)
Death	$0		≤ 24: $1,220,682^f^ 25–59: $680,621 ≥ 60: $72,463	Gu and Wong (2010)	− 1	

^a^Costs reported in 2015 Canadian dollars

^b^Acute disutility; all IMD complications are assumed to have lifelong disutility

^c^20% of lifetime productivity loss

^d^Unilateral cochlear implant cost (surgical, preoperative, and postoperative) with 25-year time horizon

^e^100% of lifetime productivity loss

^f^Lifetime productivity loss

Vaccination costs include cost of the vaccine and an administration cost of $10.10 per dose for a 2-dose schedule (De Wals et al. 2007). The vaccine price used in the analysis ($156.44 for the 2-dose series) is approximate as prices of publicly funded vaccines in Canada are confidentially negotiated with provinces through bulk procurement programs. There are currently no public contracts in place for the MenB-FHbp vaccine; hence, this value is not a negotiated and approved contract price.

A one-time quality-of-life loss, or disutility of 0.0317, is assumed to occur with each case. In addition, complicated IMD cases are assumed to accrue additional lifelong utility loss.

### Sensitivity analyses

Univariate sensitivity analyses were conducted varying the vaccination costs, vaccine uptake, carriage and disease protection against disease, and epidemiology. Each of these parameters was increased and decreased by 20% to assess the relative impact of changes on the model outcomes. Sensitivity analyses were performed for each of the 3 vaccine strategies.

## Results

Incremental cost-effectiveness ratios (ICERs) were calculated for each of the 3 vaccination strategies compared with no vaccination (Table 4). Without vaccination, 3974 MnB cases would be expected in Canada, resulting in 256 deaths and total disease-related costs of nearly $235 million over the 30-year time horizon. With 75% uptake, a routine MenB-FHbp vaccination program for 14-year-olds would yield an ICER of $975,954 per quality-adjusted life-year (QALY) and prevent 688 cases and 33 deaths, while vaccination of 17-year-olds with the same uptake would have an ICER of $684,654/QALY compared with no vaccination and could prevent an additional 345 cases and 22 deaths. If vaccination uptake among 17-year-olds were reduced to 30%, this would result in an ICER of $489,700/QALY and would prevent 575 cases and 30 deaths.

**Table 4:** Incremental cost-effectiveness over 30 years of routine adolescent MenB-FHbp vaccination strategies

	No vaccine	Strategy 1	Strategy 2	Strategy 3
		Vaccination at age 14 with 75% uptake	Vaccination at age 17 with 75% uptake	Vaccination at age 17 with 30% uptake
Total disease cases	3974	− 688	− 1033	− 575
Without complications	3124	− 541	− 809	− 451
With complications	594	− 115	− 168	− 94
Total deaths	256	− 33	− 55	− 30
Total costs	$234,870,873	$963,462,826	$1,035,258,328	$404,008,939
Direct costs	$43,422,199	$1,001,609,686	$1,093,091,508	$435,775,514
Productivity costs	$191,448,675	− $38,146,860	− $57,833,180	− $31,766,575
Vaccination costs	$0	$1,008,367,723	$1,103,135,636	$441,254,255
Life-years lost	12,461	− 2140	− 3538	− 1945
QALYs lost	6017	− 987	− 1512	− 825
Cost per QALY saved		$975,954	$684,654	$489,700

*ICER* incremental cost-effectiveness ratio, *QALY* quality-adjusted life-year

**Fig. 3:**
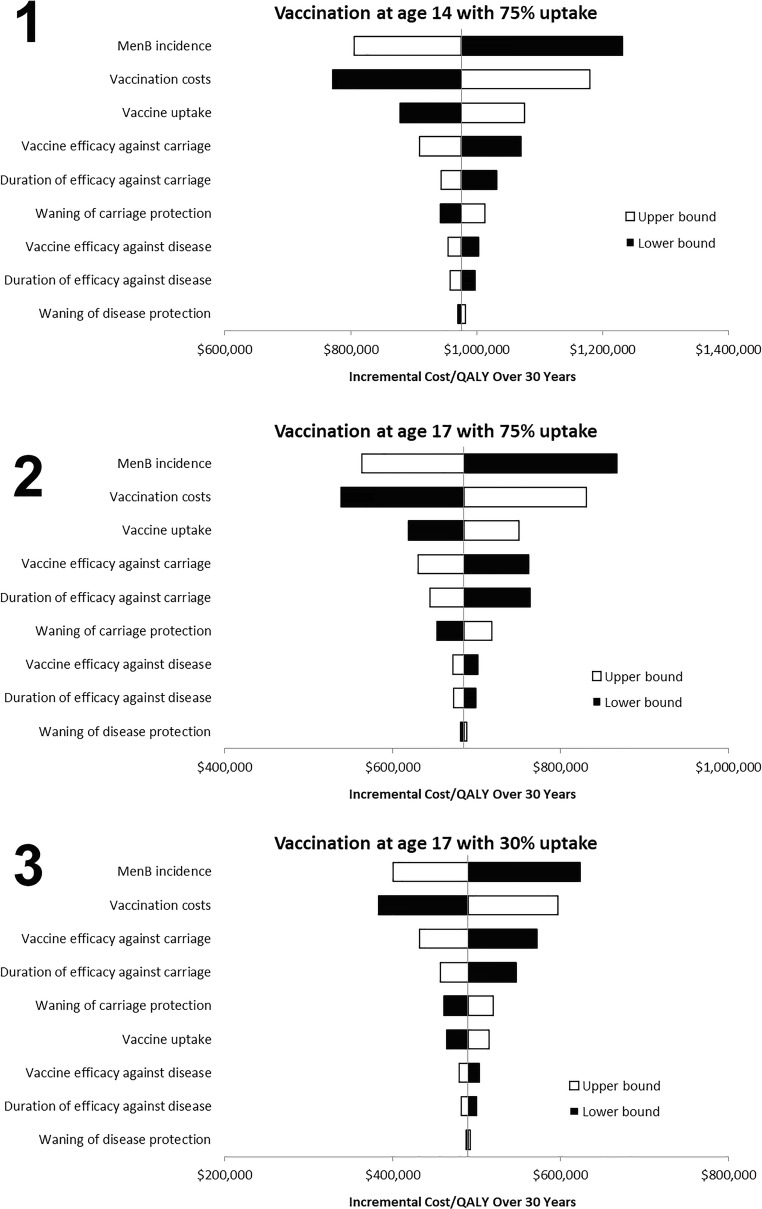
Univariate sensitivity analyses of key parameters for vaccination. Strategy 1 (age 14 with 75% uptake), strategy 2 (age 17 with 75% uptake), and strategy 3 (age 17 with 30% uptake). *ICER* incremental cost-effectiveness ratio, *QALY* quality-adjusted life-year. The centre of each plot corresponds to the base case ICER obtained for each of the 3 scenarios (Table 4). The horizontal bars represent the ICER with each parameter 20% higher (white bars) or 20% lower (black bars) than the base case

Results of univariate sensitivity analyses are similar for the 3 immunization strategies (Fig. 3). With an assumption of 75% vaccine uptake among 14- and 17-year-olds, the top 5 most sensitive variables in the model were disease incidence, vaccination costs, vaccine uptake rate, vaccine efficacy against carriage, and vaccine duration of protection against carriage or waning carriage protection, whereas vaccine efficacy against carriage was the third most sensitive variable in the 30% uptake at age 17 strategy, higher than vaccine uptake. Of note, increases in vaccine uptake result in higher ICERs due to the impact of indirect immunity. That is, additional vaccination in unvaccinated individuals incurs the full costs of vaccination but accrues proportionally smaller additional disease benefits because those who were unvaccinated already had some benefit via indirect protection. Results of scenarios analyses are presented in Table 5.

**Table 5:** Scenario analyses with alternate model assumptions

Scenario	Cost/QALY over 30 years ($)		
	Age 14, 75% uptake	Age 17, 75% uptake	Age 17, 30% uptake
Base case	975,954	684,654	489,700
1.5% discounting	637,748	446,309	314,785
20% increase in MnB incidence	805,714	563,063	400,560
2011–2016 surveillance incidence^a^	1,300,447	1,041,184	738,366
No vaccine protection against carriage	3,362,455	2,916,604	2,916,604
Alternate mixing matrix^b^	536,468	411,504	316,176

*QALY* quality-adjusted life-year

^a^Age-specific surveillance incidence data provided by the Public Health Agency of Canada (Public Health Agency of Canada n.d.)

^b^Assumes broader carriage transmission among age groups than the base case, which assumes 98% of transmission occurs within a 3-year age range

## Discussion

Our model demonstrates that routine adolescent MenB-FHbp vaccination could have a substantial public health impact in Canada, although it did not reach commonly accepted cost-effectiveness thresholds (Walker et al. 2010). Even at less than half the vaccine uptake (30% vs 75%), vaccinating older adolescents (age 17 vs age 14) against MnB is associated with improved health outcomes and lower costs per QALY. This finding is due to age-related differences in disease incidence and carriage prevalence. At age 14, MnB protection begins during a period of relatively low incidence (0.23 cases/100,000) and decreases each subsequent year just as incidence increases to 0.73 cases per 100,000 for ages 15 through 19. In contrast, vaccination at age 17 is assumed to provide optimal protection during a period in which the incidence (0.43 cases/100,000 for ages 20 through 24) and expected carriage are higher than at age 14. For this reason, immunization at 17 years old may be a better option to provide optimal protection as well as cost-effectiveness, though achieving high immunization rates in that age group would likely constitute a challenge.

The relatively low incidence of MnB in Canada (ranging from 0.2 to 0.3 cases per 100,000 population between 2011 and 2015 (Public Health Agency of Canada 2017)) may make routine vaccination less acceptable from a cost-effectiveness perspective, relative to more common but less severe diseases. However, decision-makers should also consider and prioritize preventing the significant morbidity or mortality associated with diseases such as MnB. Black (2013) illustrated how the use of cost-effectiveness analyses alone could lead to biased favourable decisions toward vaccines that provide economic benefit rather than those that reduce severe morbidity and mortality, such as vaccines against IMD.

The most significant limitations to the current model are the lack of data to estimate meningococcal transmission and the uncertainty of the vaccine efficacy against carriage. There is very limited information on the prevalence of *N. meningitidis* carriage in general, so carriage estimates for our model were calculated using MnB and MnC studies in a UK population. The longitudinal study of 2010 to 2013 MnB bacteria throat carriage prevalence among 13- to 25-year-olds in Quebec (Gilca et al. 2018) was not available at the time of our analysis. Nevertheless, our estimates of carriage prevalence in the 15 to 19 years age group were similar to those reported by Gilca et al. for 9th and 11th graders. We adapted the UK-based MnC population mixing matrix from Trotter et al. (2005) for estimating MnB-specific transmission data. Similar to our findings, results of other published health economic evaluations of MnB vaccines were shown to be sensitive to assumptions surrounding disease incidence, mortality, and vaccine protection against carriage (Black 2013; Drummond et al. 2007; Getsios et al. 2004; Kauf 2010). Hence, small changes in the model assumptions regarding disease and carriage could result in potentially different conclusions. However, we believe the current results are conservative estimates of the benefits of adolescent vaccinations as our assumptions regarding vaccine uptake, indirect costs, and effectiveness against carriage were lower compared with those reported in other published 4CMenB economic analyses (Pan-Canadian Public Health Network et al. 2014).

With regard to discounting, the UK’s National Institute for Health and Care Excellence (NICE) and the Canadian Agency for Drugs and Technologies in Health (CADTH) both recommend the use of a 1.5% discount rate for costs and benefits (Canadian Agency for Drugs and Technologies in Health 2017), as higher discounting rates could undervalue the benefit of preventive and public health projects that have large costs up front but accrue benefits over decades (Rawlins et al. 2010). Using a 1.5% discount rate in our model yielded 35% lower ICER ratios compared with the 3% used for the base case.

The results of this study are in line with previous cost-effectiveness model studies conducted mainly for infant MnB vaccination in Canada and Europe, which also resulted in ICERs beyond current acceptability thresholds. Tu et al. (2014) conducted an economic evaluation of infant MnB vaccination in Ontario using higher efficacy assumptions (90% vaccine effectiveness, 66% strain coverage, 97% vaccine coverage) than our model, and concluded that the program was unlikely to be considered economically attractive in Canada. Although vaccinating a cohort of 150,000 infants could be expected to prevent 4.6 MnB IMD cases and 0.5 related deaths over the cohort’s lifetime, cost savings from prevented cases could not offset the vaccination program cost, resulting in an ICER of CAD $4.76 million per QALY gained.

Traditional health economic evaluations do not appear to capture the full impact of a MnB vaccination program. Analyses in The Netherlands (Pouwels et al. 2013), Italy (Gasparini et al. 2016), England (Christensen et al. 2013), and France (Lecocq et al. 2016) all found an infant immunization program not to be cost-effective. However, when the authors of the English study included more “favourable” assumptions, including quality-of-life losses for family and network members, 1.5% discounting, and the value of litigation costs, results showed that a routine infant vaccination could be cost-effective with a lower vaccine price (Christensen et al. 2016). In our analysis, in order to obtain a cost per QALY of $135,000 (considered the highest limit of cost-effectiveness according to the WHO guidelines (Walker et al. 2010)), the disease incidence would have to be 4.65 times higher (even more if using the most recent data (Public Health Agency of Canada 2017)) or a vaccine price of $11 per dose.

## Conclusion

MnB disease is rare but potentially devastating and life-threatening. A significant number of cases occur in adolescents and young adults, who are also at higher risk of carrying *N. meningitidis* and transmitting the disease. Although the implementation of routine adolescent MenB-FHbp vaccination could have a substantial public health impact in preventing IMD cases and deaths, given the current low incidence of MnB disease in Canada, our results show that such a program would not be cost-effective. As cost-effectiveness plays a significant role in the current evaluation framework of new vaccines in Canada, the potential for recommendation and funding of an immunization program with an ICER beyond acceptable thresholds is low. However, IMD incidence has been historically unpredictable; other criteria such as prevention of outbreaks, peace of mind for parents and society, ethical considerations, and the potential positive impact of vaccination on antimicrobial resistance should be considered in the decision-making.

## Footnotes

1 At the time of manuscript writing, NACI had not yet issued its recommendation for MenB-FHbp.
